# Mechanical Valves: Past, Present, and Future—A Review

**DOI:** 10.3390/jcm13133768

**Published:** 2024-06-27

**Authors:** Dror B. Leviner, Dana Abraham, Tom Ronai, Erez Sharoni

**Affiliations:** 1 Department of Cardiothoracic Surgery, Carmel Medical Center, Haifa 3436212, Israel; danaab@campus.technion.ac.il (D.A.); tomronai@campus.technion.ac.il (T.R.); esharoni@clalit.org.il (E.S.); 2 The Ruth & Baruch Rappaport Faculty of Medicine, Technion, Haifa 3525433, Israel

**Keywords:** mechanical valve, aortic-valve replacement, mitral-valve replacement, bioprosthetic valve

## Abstract

The mechanical valve was first invented in the 1950s, and since then, a wide variety of prostheses have been developed. Although mechanical valves have outstanding durability, their use necessitates life-long treatment with anticoagulants, which increases the risk of bleeding and thromboembolic events. The current guidelines recommend a mechanical prosthetic valve in patients under 50–60 years; however, for patients aged 50–70 years, the data are conflicting and there is not a clear-cut recommendation. In recent decades, progress has been made in several areas. First, the On-X mechanical valve was introduced; this valve has a lower anticoagulant requirement in the aortic position. Second, a potential alternative to vitamin K-antagonist treatment, rivaroxaban, has shown encouraging results in small-scale trials and is currently being tested in a large randomized clinical trial. Lastly, an innovative mechanical valve that eliminates the need for anticoagulant therapy is under development. We attempted to review the current literature on the subject with special emphasis on the role of mechanical valves in the current era and discuss alternatives and future innovations.

## 1. Introduction

In the United Sates alone, isolated surgical aortic-valve replacement (SAVR) is performed in around 18,000 patients and isolated mitral-valve replacement (MVR) is performed in around 11,000 patients each year [1]. During surgery, the native valve is resected and a prosthetic valve is implanted instead. The prosthetic valve can be either bioprosthetic or mechanical, and each type carries its own advantages and risks. The key limitation of a bioprosthetic valve is the higher rate of reintervention due to structural valve deterioration (SVD), while the use of a mechanical valve requires ongoing treatment with anticoagulants, which increases the risk of bleeding and thromboembolic events. The current guidelines are very clear about prosthetic choice in patients younger than 50 and older than 70 years, but in between, there is a “grey zone”, which requires shared decision-making between the patient and the surgeon [2,3].

Since the first successful mechanical-valve implantation in 1952 [4], mechanical valves have continually improved to have better hemodynamic performance and less need for intensive anticoagulant treatment. Simultaneously, several approaches were introduced as an alternative to surgical valve replacement, such as transcatheter valve interventions. In this article, we aim to review the role and management of mechanical valves in the current era, including the risks and benefits of mechanical valves, current guidelines on prosthetic choice, alternatives, and future directions. We conducted a comprehensive review of the current literature using PubMed and addressed each relevant article including meta-analyses, clinical trials, and technology advances.

## 2. History and Trends

**Figure 1:**
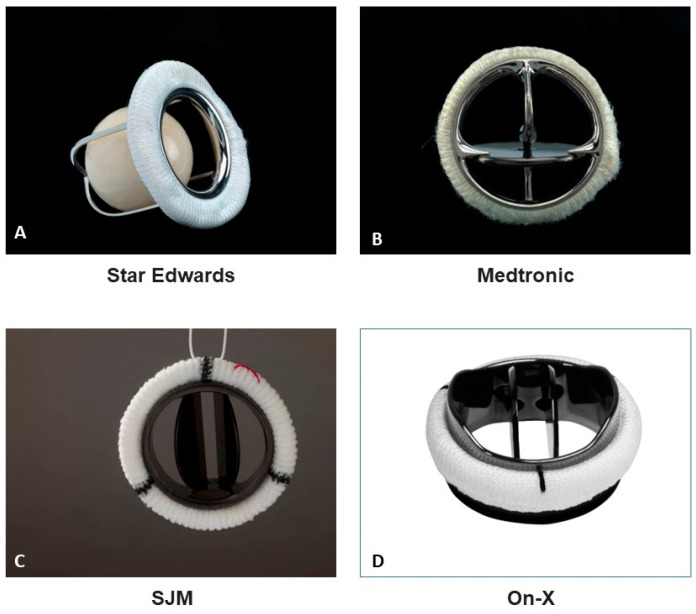
Mechanical prosthetic valve types. (**A**) Star Edwards ball in a cage; (**B**) Medtronic Hall tilting disc; (**C**) St. Jude medical bileaflet disc; (**D**) On-X bileaflet disc. Pictures obtained with permission from the Division of Medicine and Science, National Museum of American History, Smithsonian Institution, and from Artivion.

The first generation of mechanical valves was the caged ball, which was introduced in the 1960s by Starr, Braunwald and Harkin and was used in both the aortic and mitral positions. Some of those valves were durable for up to 40 years, but they required intense use of anticoagulants [5,6,7]. Due to compromised hemodynamic performance and abnormal flow patterns, the second generation of mechanical valves, the tilting disc, was developed and introduced in the 1970s. Due to their low profile, they were easier to implant, but they were not without problems such as stasis, sticking and embolization, and, therefore, the Bjork-Shiley valve was discontinued [8]. In the early 1980s, the third generation of mechanical valve was introduced by St. Jude medical (SJM): the bileaflet prosthesis. Those valves presented better hemodynamic performance and lower rates of thromboembolism; therefore, the recommendations for anticoagulants were less intensive than those for previous models [9,10,11]. Moreover, they were easier to implant than previous generations and were preferred by most surgeons to such an extent that Medtronic ceased production of the Medtronic Hall tilting-disc prosthesis despite its good performance [12]. Following the introduction of the SJM prosthesis, several other bileaflet models entered the market, such as CarboMedics, ATS Medical and the On-X prosthesis (Figure 1). The design and configuration of those models have been changed and improved over the last three decades to allow the implantation of larger valve sizes, better hemodynamic performance, better mimicking of the normal flow pattern and, most importantly, reduction in the target INR.

**Figure 2:**
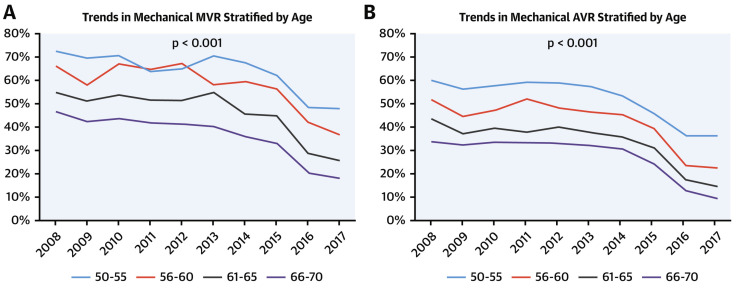
Trends in mechanical-valve use among patients aged 50 to 70 years. (**A**) Mitral-valve replacement (MVR); (**B**) Aortic-valve replacement (AVR). Reproduced with permission from Alkhouli et al. [14].

Despite the improvements and better long-term results attained with mechanical valves over time, their use in both the mitral and aortic positions has decreased over the last few decades. Although this decline was mostly noted among patients ages 65 to 70 years, it was consistent across all ages (Figure 2) [13,14,15,16,17,18]. There are several possible contributing factors to this decline. First, the fear of anticoagulant therapy with warfarin. To prevent thromboembolic complications, patients with mechanical valves must take warfarin and monitor the International Normalized Ratio (INR) closely. Due to the prolonged INR, those patients are at an increased risk for bleeding, especially at high INR values. Another possible explanation is the improvement in bioprosthetic valves over the last two decades, which have shown greater durability in the third generation and a lower incidence of SVD [19,20,21,22]. In addition, the introduction of Transcatheter Aortic Valve Implantation (TAVI) dramatically changed the incidence of SAVR and thus also contributed to the decline in the usage of mechanical prosthesis [1,23,24]. Moreover, the possibility of valve-in-valve TAVI for failed bioprosthetic valves offers an alternative solution for patients with suitable anatomy who wish to avoid anticoagulants, although this is not suitable for all patients, especially those in whom a smaller valve was implanted in surgery [25].

## 3. Current Guidelines

There are two major guidelines regarding the management of valvular heart disease: those published by the American College of Cardiology/American Heart Association (ACC/AHA) and those published by the European Society of Cardiology/European Association of Cardiothoracic Surgery (ESC/EACTS) [2,3].

Both ACC/AHA and ESC/EACTS agree that the choice of prosthetic valve should be based on the desire of the informed patient if there is no contraindication to anticoagulants and the therapy can be managed appropriately (Class I, Level C) (Table 1). In patients who require SAVR, it is reasonable to choose a mechanical prosthetic valve in patients younger than 50 years old according to the ACC/AHA or in patients younger than 60 years old according to the ESC/EACTS (Class 2a, Level B). For patients who require SAVR and are between 50 and 65 years old, according to ACC/AHA, or between 60 and 65 years old, according to ESC/EACTS, the choice should be made with consideration of the individual patient’s factors and a process of shared decision-making, which puts these patients in the so called “gray zone” (Class 2a, Level B). In patients older than 65 years old who require SAVR, it is reasonable to choose a bioprosthetic valve, according to both the ACC/AHA (Class 2a, Level B) and the ESC/EACTS (Class 2a, Level C). For patients younger than 65 years who require MVR, it is reasonable to choose a mechanical prosthetic valve, according to both guidelines (Class 2a, Level B). In patients older than 65 years old, according to ACC/AHA, or older than 70 years old, according to ESC/EACTS, it is reasonable to choose a bioprosthetic valve (Class 2a, Level B). The ESC/EACTS also recommends a mechanical prosthetic valve in patients who are at high risk of accelerated SVD because of factors such as young age (<40 years), hyperparathyroidism and use of hemodialysis (Class 1, Level C).

## 4. Clinical Outcomes of Mechanical vs. Bioprosthetic Valve

### 4.1. Survival

Most of the relevant studies have shown a survival benefit for mechanical valves over bioprosthetic valves in patients younger than 70 years undergoing SAVR and patients younger than 65 years undergoing MVR [16,26,27,28,29,30,31,32,33]. Three randomized controlled trials (RCTs) compared long-term survival in patients who received mechanical vs. bioprosthetic valves. Two of these studies, the Veterans Affairs Randomized Trial and the study by Oxenham et al., compared patients who underwent either SAVR or MVR, and the study by Stassano et al. compared patients who underwent isolated SAVR. The Veterans Affairs Randomized Trial showed a lower incidence of all-cause 15-year mortality for patients who received a mechanical aortic valve over a bioprosthetic aortic valve (66% vs. 79%, *p* = 0.02), but no difference in all-cause 15-year mortality in patients who underwent MVR (81% vs. 79%, *p* = 0.3) [33]. The study by Oxenham et al. showed no difference in survival over 20 years of follow-up between mechanical and bioprosthetic valves (25% vs. 22.6%, *p* = 0.39) but better survival without reoperation in both the mitral and aortic positions with mechanical valves (23.5% vs. 6.7%, *p* < 0.0001) and a better survival without a major event in patients who had received a mechanical MVR vs. bioprosthetic MVR (13.8% vs. 4.8%, *p* = 0.0007) [34]. The study by Stassano et al. showed similar overall mortality rates between patients between 55 and 70 years old who underwent AVR with either a mechanical or bioprosthetic valve (27.5% vs. 30.6%, *p* = 0.6) [35].

Various studies in the last decade have concluded that implantation of a bioprosthetic aortic valve is a reasonable choice in patients aged 50 to 65 due to the lack of survival benefit associated with a mechanical aortic valve [16,36,37,38,39,40,41]. On the other hand, several recent studies showed a survival benefit for aortic mechanical prosthesis in patients younger than 65–70 years old. The AUTHEARTVISIT, a population-based cohort study of 13,993 patients who underwent isolated SAVR, showed that the use of bioprosthetic valves was associated with a higher mortality rate in patients aged 50 to 65 years (hazard ratio (HR) 1.676, 95% CI: 1.289–2.181, *p* < 0.001) [28]. Kyto et al. conducted a retrospective analysis with propensity-score matching (PSM) of 576 pairs aged 50 to 70 years old who underwent SAVR with a mean follow up of 6.7 years and showed a lower long-term mortality rate in patients who received mechanical valves (HR 0.72, 95% CI: 0.54–0.97, *p* = 0.028) [27]. An HR meta-analysis of RCTs and PSM or inverse probability weighting (IPW) observational studies examined the clinical outcomes of patients between 50 and 70 years who underwent SAVR. It concluded that patients aged 50 to 70 who received a mechanical aortic valve had a significant survival benefit compared to patients who received bioprosthetic aortic valves (HR 1.22, 95% CI: 1.00–1.49) [29]. Another independent HR meta-analysis of patients younger than 70 years old who underwent AVR came to the same conclusion (HR 0.76, 95% CI: 0.7–0.83, *p* < 0.0001) [26].

Studies on long-term survival after MVR in the last decade have also shown contradictory results. Chikwe et al. conducted a retrospective analysis with PSM of 664 pairs of patients aged 50 to 69 with a median follow-up of 8.2 years and found no survival difference between mechanical valves and bioprosthetic valves (HR 0.95, 95% CI: 0.79–1.15) [42]. Nishida et al. evaluated long-term mortality in Japanese patients who underwent MVR and found a survival benefit for bioprosthetic valves in patients older than 70 (*p* = 0.0139) and similar survival rates for patients younger than 70 years old [43]. Schnittman et al. conducted a retrospective analysis with PSM of 373 paired patients aged 18 to 50 who underwent MVR with a median follow-up of 12.4 years and found better long-term survival rates for mechanical mitral valves (HR 1.67, 95% CI: 1.21–2.32, *p* = 0.002), especially in patients aged 41–50 years old (HR 1.98, 95% CI: 1.34–2.94, *p* < 0.001) [44]. Goldstone et al. compared long-term outcomes between IPW cohorts of patients who underwent primary SAVR or MVR with mechanical or bioprosthetic valves and showed higher mortality rates in patients younger than 70 who received bioprosthetic valves in the mitral position (40–49 years old—HR 1.88, 95% CI: 1.35–2.63, *p* < 0.001, 50–69 years old—HR 1.16, 95% CI: 1.04–1.30, *p* = 0.01, respectively) [16]. Leviner et al. conducted a retrospective analysis with PSM-adjusted analysis of 208 paired patients aged 50–70 who underwent MVR with a median follow up of 66.6 months and showed a greater risk for mortality in patients aged 50 to 64 years who received bioprosthetic mitral valves (HR 1.5, 95% CI: 1.07–2.1, *p* = 0.018) [31]. Chen et al. evaluated long-term survival in patients with rheumatic heart disease who underwent MVR using PSM, which resulted in 788 pairs, and found higher rates of all-cause mortality in patients who received a bioprosthetic mitral valve up to the age of 65 (HR 1.19, 95% CI: 1.01–1.41, *p* = 0.04) [32].

In conclusion, recent studies are inconclusive regarding the survival benefit of mechanical prosthesis in patients aged 50 to 65 years old in either the mitral or the aortic position (Table 2).

### 4.2. Reoperation

The risk of reoperation is higher in patients who undergo either AVR or MVR with a bioprosthetic valve [16,26,29,30,32,34,35,38,39,40,41,42,43,44,45]. The Veterans Affairs Randomized Trial showed higher rates of reoperation in patients who received bioprosthetic aortic valves (29% vs. 10%, *p* = 0.004); however, the rate of reoperation in patients who underwent MVR was not significantly different between the two types of prosthetic valves (50% vs. 25%, *p* = 0.15) [33]. The AUTHEARTVISIT trial showed a higher reoperation rate for patients who underwent SAVR with bioprosthetic valves in both patients younger than 50 and patients aged 50–65 (HR 3.511, 95% CI: 1.24–9.938, *p* = 0.02, HR 3.483, 95% CI: 1.445–8.396, *p* < 0.01, respectively) but similar rates of reoperation in patients older than 65 (HR 0.569, 95% CI: 0.204–1.584, *p* = 0.28) [28]. Kyto et al. also showed lower rates of reoperation in patients who received mechanical aortic valves (HR 0.3, 95% CI: 0.12–0.74, *p* = 0.009), and an increasing rate of reoperation during the follow-up period in the bioprosthetic group, from 1.3% in the first year to 8.5% in 10 years, with a 23.1% perioperative mortality (which is a higher than is reported in most of the literature) in reoperation [27]. Chiang et al. conducted a retrospective analysis with PSM of 1001 pairs of patients who underwent AVR with either a mechanical or a bioprosthetic valve. They concluded that the risk of reoperation was significantly lower in the mechanical group (HR 0.52, 95% CI: 0.36–0.75) and that the 30-day mortality after reoperation was 9% in the cohort [36].

Redo valve replacement is considered complex surgery with higher rates of mortality and morbidity compared to primary valvular surgery. Mehaffey et al. evaluated all mitral-valve cases in the regional database of the Society of Thoracic Surgery (STS) of Virginia between 2002–2016 and compared outcomes of patients who underwent primary mitral-valve surgery (n = 10,145) to patients who underwent redo mitral-valve surgery (n = 1096). The operative mortality in redo MVR was significantly higher in the redo group compared to the primary group (11.1% vs. 6.5%, *p* < 0.0001), as were the length of stay, ventilation time and rates of reoperation, stroke and renal failure [46]. Ejiofor et al. presented survival outcomes in 247 patients who underwent redo MVR and reported an operative mortality rate of 9.3% [47]. Kaneko et al. conducted a retrospective analysis of the STS database in patients who underwent redo SAVR after primary SAVR between 2011–2013 (n = 3380) and compared outcomes to patients who underwent primary SAVR in the same time period (n = 54,183). Redo SAVR had higher rates of operative mortality (4.6% vs. 2.2%, *p* < 0.0001), stroke (1.9% vs. 1.4%, *p* = 0.02), renal failure (4.2% vs. 2.5%, *p* < 0.0001) and reoperation for bleeding (3.9% v.s 3.2%, *p* = 0.028) [48]. Narayan et al. conducted a retrospective nationwide analysis in the UK with PSM of 3015 paired patients who underwent isolated redo SAVR compared to patients who underwent isolated primary SAVR. Redo SAVR had higher rates of operative mortality (3.1% vs. 1.9%, *p* < 0.001) and cerebrovascular accident (CVA) (2.1% vs. 1.5%, *p* < 0.001) [49]. Mahboubi et al. conducted a retrospective analysis with PSM of 581 paired patients who underwent isolated redo SAVR compared to isolated primary SAVR. They also showed higher rates of in-hospital mortality in the redo group (2.4% vs. 0.69%, *p* = 0.02); however, the rate of mortality in the redo group decreased over the years, from 3.4% in 1985 to 1.3% in 2011 [50].

In summary, the risk of reoperation is greater with bioprosthetic valves in both the mitral and aortic positions, and reoperation is associated with increased risk for mortality and morbidity.

### 4.3. Bleeding

The risk of bleeding is higher in patients who undergo either SAVR or MVR with a mechanical valve [16,26,29,30,33,34,38,39,40,42]. Stassano et al. showed a comparable risk for bleeding in patients aged 55 to 70 years who underwent AVR with either a mechanical or a bioprosthetic valve (*p* = 0.08) [34]. Rodríguez-Caulo et al. conducted a multicenter observational study with PSM and compared long-term outcomes in patients with severe aortic stenosis who underwent SAVR with either a mechanical or a bioprosthetic valve and showed a higher risk of bleeding in the mechanical group (HR 0.65, 95% CI: 0.49–0.87, *p* = 0.004); however, in patients younger than 55 years, there were no difference in the risk of bleeding (HR 1.043, 95% CI: 0.391–2.777, *p* = 0.93) [41]. The AUTHEARTVISIT study showed that the type of aortic prosthetic valve was not associated with major bleeding other than intracranial hemorrhage (HR 0.873, 95% CI: 0.436–1.747, *p* = 0.7) [45]. Kyto et al. also showed no interaction between the prosthesis type in the aortic position and major bleeding, including intracranial hemorrhage (HR 1.19, 95% CI: 0.79–1.8, *p* = 0.402) [27]. Chen et al. showed comparable rates of bleeding in patients who underwent MVR with either a mechanical or a bioprosthetic valve (HR 0.99, 95% CI: 0.79–1.24, *p* = 0.919) [32]. Chiang et al. demonstrated higher rates of major bleeding with a mechanical valve (HR 1.75, 95% CI 1.27–2.43) and found that the 30-day mortality after a major bleeding event in the cohort was 13.2% [36]. Nishida et al. observed no difference in bleeding in patients who underwent MVR (*p* = 0.275); however, in a subgroup analysis, there was a higher rate of major bleeding in patients younger than 65 years who received a mechanical mitral valve (*p* = 0.0496) [43]. Schnittman et al. showed a similar risk-adjusted cumulative incidence of major bleeding in patients who underwent MVR with either a mechanical or bioprosthetic valve (HR 0.78, 95% CI: 0.46–1.32) [44].

To conclude, most of the studies attribute a higher risk for major bleeding to mechanical valves. However, several recent studies showed a comparable risk between the two types of prosthetic valve.

### 4.4. Stroke

The majority of studies found that neither type of prosthetic valve is associated with a greater risk of stroke [26,27,28,29,30,31,32,36,37,38,39,40,41,44,45]. However, two recent studies showed a higher incidence of stroke among patients who received mechanical valves. Chikwe et al. demonstrated a higher incidence of stroke among patients who received a mechanical mitral valve compared to those who received a bioprosthetic valve (HR 1.62, 95% CI 1.1–2.39) [42]. Goldstone et al. showed higher cumulative incidence of stroke among patients aged 45 to 54 who received a mechanical aortic valve (HR 0.64, 95% CI: 0.46–0.86) and in patients aged 50 to 69 who received a mechanical mitral valve (HR 0.83, 95% CI: 0.72–0.97) [16].

## 5. Anticoagulants

The prominent drawback of mechanical valves is the lifelong need for anticoagulant therapy due to the thrombogenicity of the prosthetic material and design. The consequences of undertreatment can be serious, including events such as valve thrombosis or major thromboembolic events [51,52,53]. The risk of a thromboembolic event is higher for a mechanical prosthetic valve in the mitral position than for one in the aortic position [51,54]. However, the risk of a thromboembolic event declined significantly from the first-generation the ball-in-cage valve to the next-generations tilting-disc and bileaflet valves [55].

The only currently approved long-term anticoagulant therapy for mechanical valves is a vitamin K antagonist (VKA). Treatment is usually initiated on the first postoperative day in combination with bridging therapy with either unfractionated heparin (UFH) or low-molecular-weight heparin (LMWH) until therapeutic INR is achieved. Historically, the recommended target INR was between 3 and 4.5 for all kinds of mechanical prosthetic valves [56]. In the 1990s, several RCTs demonstrated lower bleeding risk with no significant increase in the risk of thromboembolic events at a lower target INR of 2.0–3.0 in both AVR and MVR with a mechanical valve [57,58,59]. More recent studies on mechanical aortic valves showed similar results: decreased risk of bleeding with similar incidence of thromboembolic events at a target INR of 1.5–2.5 and 1.5–2.0 [60,61]. Many studies searched for an alternative to VKA due to the increased risk of bleeding, the complex management, and the difficulty of maintaining patients in the therapeutic range. Some of them focused on antiplatelet therapy and others on direct-oral-anticoagulants (DOACs) therapy. Mok et al. conducted an RCT of 254 patients who received 1st- or 2nd-generation mechanical valves in either the aortic or mitral position or in both positions and who were randomized into three groups: the first received VKA; the second, dipyridamole-aspirin and the third, pentoxifylline-aspirin. The rate of thromboembolic events was significantly lower in the VKA group compared to the other groups, but the rate of bleeding was significantly higher [62]. A meta-analysis of 11 RCTs evaluated the benefit of adding an antiplatelet therapy to VKA and concluded that the addition of antiplatelet therapy reduced the risk of thromboembolic events by 57% while increasing the risk of major bleeding by 58% [63]. Another RCT by Puskas et al. tested whether 576 patients with no thromboembolic risk factors could be safely managed with dual-antiplatelet therapy (DAPT) of clopidogrel plus aspirin compared to standard-dose warfarin plus aspirin. The study was terminated prematurely due to excess cerebral thromboembolic events in the DAPT group at 8.8 years of follow-up [64]. Regarding DOACs, dabigatran is contraindicated in patients with mechanical prosthetic valves. The RE-ALIGN randomized trial was terminated prematurely after enrolling 252 patients due to an excess of thromboembolism and bleeding events in the dabigatran group [65]. The PROCAT-XA randomized trial evaluated the efficacy and safety of apixaban compared to warfarin in the setting of mechanical SAVR using the On-X valve. The trial was discontinued early after enrolment of 863 patients owing to an increased rate of valve thrombosis and thromboembolic events in the apixaban cohort [66]. The RIWA randomized trial assessed the incidence of thromboembolic and bleeding events associated with rivaroxaban vs. warfarin in patients who underwent either MVR or AVR and concluded that in short-term follow-up of 3 months, the clinical outcomes were similar between the groups [67].

A major improvement in the anticoagulant therapy was first observed in a poorly anticoagulated population in South Africa. Williams et al. evaluated the clinical performance of the On-X valve among patients who underwent MVR or SAVR or double-valve surgery. Among the patient population, 40% were either not anticoagulated or unsatisfactorily anticoagulated. At 4 years of follow-up of 438 patients, there was only one case of valve thrombosis in the mitral position, and the linearized rate of thrombosis was 0.0% per patient year after SAVR or double-valve surgery and 0.2% per patient year after MVR [68]. This demonstration of a low incidence of valve thrombosis in a real-world setting with the On-X prosthetic valve set the path for the PROCAT trial. The trial randomized 375 patients with one or more thromboembolic risk factors to undergo SAVR with the On-X valve and receive either lower-dose warfarin plus aspirin to achieve a target INR of 1.5–2 or standard-dose warfarin plus aspirin to achieve a target INR of 2.0–3.0. The lower-target-INR group experienced higher rates of freedom from bleeding (90.8% vs. 77.9%, *p* = 0.002) with similar rates of freedom from thromboembolic events (89.3% vs. 93.1%, *p* = 0.2) [64]. Based on the results of this study, the FDA approved a lower target INR of 1.5–2.0, preferably with concomitant aspirin, after SAVR with the On-X mechanical prosthetic valve. Considering these promising results, investigators assessed the efficacy and safety of lower target INR, also with the On-X mitral valve, in the PROCAT mitral study. A total of 401 patients were randomized to low-dose warfarin plus aspirin with target INR of 2.0–2.5 or standard-dose warfarin plus aspirin with a target INR of 2.5–3.5. Disappointingly, they concluded that low-dose warfarin did not achieve non-inferiority compared to standard-dose warfarin for the composite primary end point of thromboembolism, valve thrombosis and bleeding [69].

Current guidelines recommend targeting a median INR value rather than a range to avoid extreme values in the range. High INR variability is an independent predictor of adverse events after valve replacement. Both the ACC/AHA and the ESC/EACTS current guidelines recommend a target INR of 2.5 for patients with a mechanical bileaflet valve in the aortic position and no risk factors for thromboembolism (Class 1, Level B). For patients with a mechanical bileaflet valve or Medtronic-Hall tilting disc in the aortic position with one or more risk factors for thromboembolism (atrial fibrillation, previous thromboembolism, left ventricular dysfunction, or hypercoagulable state) a target INR of 3.0 is recommended (Class 1, Level B). For patients with a mechanical mitral valve, a target INR of 3.0 is recommended (Class 1, Level B). For patients with an On-X mechanical aortic valve and no risk factors for thromboembolism, a target INR of 1.5–2.0 is reasonable in combination with daily aspirin (Class 2, Level B) [2,3].

## 6. Alternatives

Due to the life-long need for anticoagulant therapy and its adverse effects, several surgical alternatives to mechanical valve replacement have been developed over the years.

### 6.1. Aortic Valve

There are several surgical techniques used to repair an aortic valve in patients with pure aortic regurgitation (AR). Those techniques show acceptable long-term results, with both an overall survival of 81–87% and freedom from reoperation of 49–80% [70,71,72]. However, the data regarding the efficacy of aortic-valve repair vs. SAVR is limited, with some of the studies showing similar or better clinical outcomes and some higher rates of reintervention in the repair group [73,74,75]. Girdauskas et al. assessed 1-year clinical outcomes after SAVR vs. aortic-valve repair in 8076 patients from the German Aortic Valve Registry and showed that aortic-valve repair was associated with a significant survival advantage (HR 0.68, 95% CI 0.51–0.9, *p* < 0.001) and with a similar rate of reintervention [74]. Jabagi et al. conducted a retrospective analysis with PSM of 70 paired patients who underwent either repair or replacement of the aortic valve and compared a composite outcome of infective endocarditis, myocardial infarction (MI), stroke, transient ischemic attack (TIA), thromboembolism, bleeding and aortic-valve reoperation. The event-free survival from the composite outcome at the 10-year follow-up was significantly better after aortic-valve repair than after replacement (82% vs. 68%, *p* = 0.024), although the 10-year overall survival rate was similar (82% vs. 72%, *p* = 0.29) [75]. Patients with aortic-root aneurysms, which lead to severe AR, can benefit from valve-sparing aortic-root replacement (VSARR) surgery. VSARR has been shown to be a safe and effective alternative to the Bentall procedure (aortic-root replacement with a graft that incorporates an aortic valve) in patients with non-stenotic aortic valves [76,77,78,79,80,81,82,83]. The CAVIAAR prospective multicenter cohort study followed 261 patients, 130 of whom underwent VSARR and 131 of whom underwent a Bentall. The VSARR group showed significantly fewer valve-related deaths at 4 years (HR 0.09, 95% CI: 0.02–0.34) and fewer major bleeding events (HR 0.37, 95% CI: 0.16–0.85) without an increased risk of valve-related reoperation (HR 2.1, 95% CI: 0.64–6.96) [78]. Ouzounian et al. conducted a retrospective analysis using PSM, with a mean follow-up of 9.8 years, of 616 patients who underwent aortic-root surgery (253 VSARR patients, 180 modified Bentall patients (i.e., a Bentall with a biological valve), and 183 Bentall patients). The Bentall group was associated with increased long-term major adverse valve-related events compared to the VSARR group (HR 5.2, *p*< 0.001), increased cardiac mortality (HR 6.4, *p* = 0.003) and increased risk of bleeding (HR 5.6, *p* = 0.008) [82]. The ESC/EACTS guidelines recommend VSARR in young patients with aortic-root dilation if the surgery is performed in experienced centers and durable results are expected [3].

Another alternative to mechanical SAVR is the Ross procedure, in which the native pulmonic valve is implanted instead of the aortic valve and a homograft is implanted in the pulmonary position. Several studies compared clinical outcomes in young adults between mechanical SAVR and the Ross procedure and showed a better survival rate in patients who underwent Ross at the expense of a higher rate of reoperation [84,85,86,87,88,89,90]. Mazine et al. conducted a meta-analysis of 18 studies, including one RCT, with a median follow-up of 5.8 years and showed a survival benefit for the Ross procedure over mechanical SAVR (incidence rate ratio (IRR) 0.54, 95% CI: 0.35–0.82, *p* = 0.004), as well as lower rates of stroke (IRR 0.26, 95% CI: 0.09–0.8, *p* < 0.001) and major bleeding (IRR 0.17, 95% CI: 0.07–0.4, *p* < 0.001), but they did find higher rates of reintervention (IRR 1.76, 95% CI: 1.16–2.65, *p* = 0.007) [90]. In light of the potential benefit of the procedure, the ACC/AHA guidelines state that in patients younger than 50 years old who prefer a bioprosthetic valve and who have suitable anatomy, it is reasonable to consider the Ross procedure at comprehensive valve centers (Class 2b, Level B) [2].

In the last two decades, the popularity of TAVI as an alternative to surgery in elderly patients has grown exponentially. As a consequence of the good results of TAVI, it has been performed in younger patients as the utilization of bioprosthetic surgical aortic valves has increased [13,14,15,16,17,18,24]. Due to the limited durability of bioprosthetic valves and the fact that they are prone to SVD, the need for reintervention arises [91,92,93]. Until recent years, the solution to SVD was redo SAVR. As a result of the improvements and innovations in the transcatheter approach, the idea of valve-in-valve (ViV) implantation was born. Bleiziffer et al. performed a large-scale assessment of long-term outcomes in 1066 patients who underwent ViV due to SVD of an aortic bioprosthetic with a median follow-up of 3.9 years. The estimated survival at eight years was 38%, and freedom from reintervention at eight years was 93.2% [94]. Ahmed et al. conducted a meta-analysis of nine studies including 9127 patients who underwent ViV vs. redo SAVR and concluded that ViV was associated with decreased 30-day mortality (odds ratio (OR) 0.56, *p* < 0.0001) but no difference in 2.25-year follow-up mortality (HR 1.02, *p* = 0.86). Moreover, they found that redo SAVR had lower 30-day paravalvular leak (OR 6.82, *p* = 0.04), lower incidence of 30-day severe patient–prosthesis mismatch (PPM) (OR 3.77, *p* < 0.0001) and lower postoperative aortic-valve gradient (mean difference 5.37, *p* < 0.0001) [25]. Both the ACC/AHA and the ESC/EACTS guidelines suggest that in patients with severe heart failure symptoms caused by a bioprosthetic valve regurgitation who are at high or prohibitive risk for surgery and who have suitable anatomy, a ViV is a reasonable option when performed at a comprehensive valve center (Class 2a, Level B) [2,3]. Despite those recommendations and encouraging short-term clinical results, the data regarding long-term survival and clinical outcomes are scarce and no RCT has been performed. In the future, TAVI and improved ViV technology (either in a previous biological SAVR or in a TAVI) might obviate the need for the use of mechanical valves altogether.

### 6.2. Mitral Valve

Mitral-valve repair (MVr) is a well-established procedure and is always considered first in virtually any clinical situation in which it is possible [95,96]. Shuhaiber et al. conducted a meta-analysis of 29 studies using approximately 10,000 patients and compared long-term outcomes of patients who underwent MVr vs. MVR. They concluded that the repair group had better survival rates than the replacement group (HR 1.58, 95% CI: 1.41–1.78) and that this was true across all kinds of etiologies [97]. According to the guidelines, MVr is recommended in patients with severe primary mitral regurgitation (MR) if durable results are expected (Class 1, Level B) [2,3].

In patients with failure of a bioprosthetic mitral valve, who are at high risk for surgical reintervention, ViV implantation may be considered (Class 2b, Level B) [2,3]. The data regarding the long-term outcomes of this procedure are limited, and further analysis, including RCTs, should be carried out. However, the limited data gathered so far suggest that the procedure is safe and is not associated with high rates of complication and mortality in short-term follow-up in patients who are not candidates for repeat surgery [98,99,100]. ViV could be a potential alternative solution to the need for reoperation in bioprosthetic SVD. Assuming ViV shows good long-term results, it could impact the usage of bioprosthetic valves in younger patients.

## 7. Future Directions

### 7.1. DOACs

The pursuit of an alternative anticoagulant therapy to VKA has been unsuccessful so far in the setting of mechanical valves, and trials with both apixaban and dabigatran showed disappointing results. However, rivaroxaban presented promising results in small-scale trials, and thus, a large-scale randomized trial was started: the Long-term Anticoagulation With Oral Factor Xa Inhibitor Versus Vitamin K Antagonist After Mechanical Aortic Valve Replacement (RENOVATE). As of the time of this writing, the trial is in its enrollment phase and is estimated to be completed on the second half of 2025.

Rivaroxaban as an alternative to VKA could be a major breakthrough. First, the adherence to treatment with DOACs is higher, since there is no need for INR-guided therapy and blood tests. Second, the risk of bleeding with rivaroxaban is expected to be lower than that associated with VKA, as studies on other indications for anticoagulant therapy have shown [101,102].

### 7.2. Trileaflet Valve

Over the last two decades, the main focus in the design of next-generation mechanical valves has been to find a new design that would eliminate the need for anticoagulant therapy. Thus far, several studies have demonstrated promising results for the tri-leaflet design without the ongoing use of anticoagulants both in vitro and in animals [103,104,105]. The TRIFLO mechanical valve showed good pre-clinical results in animals compared to bileaflet mechanical valves and was granted approval for first in-human clinical trial starting in December 2023 [105,106].

A mechanical valve that does not require ongoing anticoagulant therapy is a game-changing concept that could deeply affect the treatment of valvular heart disease. The possibility of one-time intervention without an increased risk for thromboembolic or bleeding events on the one hand or reintervention on the other is the holy grail of every cardiac physician.

## 8. Conclusions

Surgical valve intervention is currently the standard of care for valvular heart disease in non-elderly patients, but it is not without challenges. In recent decades, the use of mechanical prosthetic valves has declined despite the lack of evidence to support this trend. Mechanical prosthetic valves are associated with higher risks of bleeding and thromboembolic events and require ongoing anticoagulant therapy with VKA. In contrast, bioprosthetic valves do not require anticoagulant therapy but are associated with higher rates of reintervention due to SVD. The choice between a mechanical and a bioprosthetic valve is not always straightforward, especially in patients aged 50–70 years for whom the data regarding survival benefits are still inconclusive. Despite their limitations, mechanical valves have evolved over the last few decades. The On-X mechanical valve has shown promising results in the aortic position, with lower levels of INR. Currently, rivaroxaban is under review as an alternative to conventional VKA treatment. If rivaroxaban shows non-inferiority, it can increase adherence to treatment, lower the risk of bleeding and possibly improve long-term outcomes. Moreover, the first-in-human trial with a tri-leaflet mechanical valve without the need for anticoagulant therapy recently started after showing non-inferior results in animals and in vitro trials. Thus, despite general reservations regarding mechanical valves and a decline in their usage without sufficient evidence, we believe that mechanical valves are a viable choice for valve replacement and that current efforts may potentially improve them even further.

## Figures and Tables

**Table 1:** Current guidelines for prosthesis choice.

		ACC/AHA	ESC/EACTS
AVR	Mechanical	<50 years	<60 years
	Bioprosthetic	>65 years	
	Shared decision-making	50–65 years	60–65 years
MVR	Mechanical	<65 years	
	Bioprosthetic	>65 years	>70 years
	Shared decision-making	N/I	65–70 years

AVR—aortic-valve replacement, MVR—mitral-valve replacement, ACC—American College of Cardiology, AHA—American Heart Association, ESC—European Society of Cardiology, EACTS—European Association of Cardio Thoracic Surgery.

**Table 2:** Summary of current data on outcomes of mechanical vs. bioprosthetic valves.

	Age	AVR/MVR
Survival	<50 years	Favors mechanical
	50–65 years	Inconclusive
	>65 years	Favors bioprosthetic
Reoperation	<65 years	Favors mechanical
Bleeding	All ages	Favors bioprosthetic
Stroke	All ages	No difference

AVR—aortic-valve replacement, MVR—mitral-valve replacement.

## Data Availability

No new data were created or analyzed in this study. Data sharing is not applicable to this article.
